# Supplementary material for: Improving osteoarthritis care by digital means - Effects of a digital self-management program after 24- or 48-weeks of treatment
Source: PLoS One. 2020 Mar 4;15(3):e0229783. doi: 10.1371/journal.pone.0229783 (PMC7056265; doi:10.1371/journal.pone.0229783)
Supplement: S1 Table — (PDF) [file pone.0229783.s001.pdf]

Supplementary 1

Supplementary table 1. Comparative data for the 24-week sub-sample (n=499) and excluded participants (n=1210).

Characteristic	24-week sub-sample (n=499)	Non-reporters (n=1210)	p
Age, mean (SD)	64 (9)	64 (10)	0·42[a]
Female, n (%)	372 (75)	896 (74)	0·86[b]
Knee OA, n (%)	301 (60)	722 (60)	0·83[b]
BMI, mean (SD)	27·5 (4·9)	27·2 (5·0)	0·18[a]
Baseline pain, mean (SD)	5·7 (1·8)	5·7 (2·0)	0·51[a]
Baseline function, mean (SD)	10·4 (3·7)	10·6 (3·9)	0·21[a]

[a]Independent student's t-test. [b]Fisher's Exact test. BMI=body mass index. NRS=Numeric rating scale. OA=osteoarthritis.
