# Supplementary material for: Improving osteoarthritis care by digital means - Effects of a digital self-management program after 24- or 48-weeks of treatment
Source: PLoS One. 2020 Mar 4;15(3):e0229783. doi: 10.1371/journal.pone.0229783 (PMC7056265; doi:10.1371/journal.pone.0229783)
Supplement: S1 Checklist — (DOC) [file pone.0229783.s002.doc]

STROBE Statement—Checklist of items that should be included in reports of ***cohort studies***

|  | Item No | Recommendation | Page |
| --- | --- | --- | --- |
| **Title and abstract** | 1 | (*a*) Indicate the study’s design with a commonly used term in the title or the abstract | 1* |
|  |  | (*b*) Provide in the abstract an informative and balanced summary of what was done and what was found | 2* |
| Introduction | | |  |
| Background/rationale | 2 | Explain the scientific background and rationale for the investigation being reported | 4-5* |
| Objectives | 3 | State specific objectives, including any prespecified hypotheses | 4-5* |
| Methods | | |  |
| Study design | 4 | Present key elements of study design early in the paper | 5-8* |
| Setting | 5 | Describe the setting, locations, and relevant dates, including periods of recruitment, exposure, follow-up, and data collection | 5-8* |
| Participants | 6 | (*a*) Give the eligibility criteria, and the sources and methods of selection of participants. Describe methods of follow-up | 5-6* |
|  |  | (*b*) For matched studies, give matching criteria and number of exposed and unexposed |  |
| Variables | 7 | Clearly define all outcomes, exposures, predictors, potential confounders, and effect modifiers. Give diagnostic criteria, if applicable | 7-8* |
| Data sources/ measurement | 8 | For each variable of interest, give sources of data and details of methods of assessment (measurement). Describe comparability of assessment methods if there is more than one group | *7** |
| Bias | 9 | Describe any efforts to address potential sources of bias | 7-8* |
| Study size | 10 | Explain how the study size was arrived at | 5-6, 8* |
| Quantitative variables | 11 | Explain how quantitative variables were handled in the analyses. If applicable, describe which groupings were chosen and why | 7-8* |
| Statistical methods | 12 | (*a*) Describe all statistical methods, including those used to control for confounding | 7-8* |
|  |  | (*b*) Describe any methods used to examine subgroups and interactions | 7-8* |
|  |  | (*c*) Explain how missing data were addressed | 7-8* |
|  |  | (*d*) If applicable, explain how loss to follow-up was addressed | 7-8* |
|  |  | (*e*) Describe any sensitivity analyses | N/A |
| Results | | |  |
| Participants | 13* | (a) Report numbers of individuals at each stage of study—eg numbers potentially eligible, examined for eligibility, confirmed eligible, included in the study, completing follow-up, and analysed | 8 and Figure 1* |
|  |  | (b) Give reasons for non-participation at each stage | 8 and Figure 1* |
|  |  | (c) Consider use of a flow diagram | Figure 1* |
| Descriptive data | 14* | (a) Give characteristics of study participants (eg demographic, clinical, social) and information on exposures and potential confounders | Table 1-2 |
|  |  | (b) Indicate number of participants with missing data for each variable of interest | Figure 1 |
|  |  | (c) Summarise follow-up time (eg, average and total amount) | N/A |
| Outcome data | 15* | Report numbers of outcome events or summary measures over time | 8-10* |
| Main results | 16 | (*a*) Give unadjusted estimates and, if applicable, confounder-adjusted estimates and their precision (eg, 95% confidence interval). Make clear which confounders were adjusted for and why they were included | Page 11*, Table 3 |
|  |  | (*b*) Report category boundaries when continuous variables were categorized | N/A |
|  |  | (*c*) If relevant, consider translating estimates of relative risk into absolute risk for a meaningful time period | N/A |
| Other analyses | 17 | Report other analyses done—eg analyses of subgroups and interactions, and sensitivity analyses | N/A |
| Discussion | | |  |
| Key results | 18 | Summarise key results with reference to study objectives | 12* |
| Limitations | 19 | Discuss limitations of the study, taking into account sources of potential bias or imprecision. Discuss both direction and magnitude of any potential bias | 14* |
| Interpretation | 20 | Give a cautious overall interpretation of results considering objectives, limitations, multiplicity of analyses, results from similar studies, and other relevant evidence | 15* |
| Generalisability | 21 | Discuss the generalisability (external validity) of the study results | 14-15* |
| Other information | | |  |
| Funding | 22 | Give the source of funding and the role of the funders for the present study and, if applicable, for the original study on which the present article is based | 3* |

**Note:** An Explanation and Elaboration article discusses each checklist item and gives methodological background and published examples of transparent reporting. The STROBE checklist is best used in conjunction with this article (freely available on the Web sites of PLoS Medicine at http://www.plosmedicine.org/, Annals of Internal Medicine at http://www.annals.org/, and Epidemiology at http://www.epidem.com/). Information on the STROBE Initiative is available at http://www.strobe-statement.org.

***Item 1a page 1 excerpted text:** *Improving osteoarthritis care by digital means - Effects of a digital self-management program after 24 weeks of adherence*

***Item 1b page 2 excerpted text:** *Individuals with hip and knee OA, from the register of a digital self-management program and with 0-24-week (n=499) or 0-48-week adherence (n=138), were included. The treatment effect in terms of monthly pain (NRS, 0-10 worst to best) and physical function (30-second chair stand test (30CST), number of repetitions) change were investigated using a mixed model, controlling for the effect of age, body mass index (BMI), gender and index joint.*

*Pain NRS decreased monthly by -0·43 units (95% CI -0·51, -0·35) and -0·39 units (95% CI -0·43, -0·36), and 30CST repetitions increased monthly by 0·76 repetitions (95% CI 0·64, 0·89) and 0·72 repetitions (95% CI 0·65, 0·79), for the 24- and 48-week sub-sample, respectively. There were no clinically relevant effects on the improvement of pain or function by any of the controlled factors. The lack of a control group and randomization limit our ability to explain the mechanisms of the observed results.*

***Item 2 page 4-5 excerpted text:** *Digital self-management programmes have been developed to further facilitate access to first-line treatment for OA and to aid patients in maintaining a long-term exercise regime [21, 22]. Early reports have shown reduction in pain, disability and desire for surgical treatment in patients with OA after six weeks in a digital self-management programme [23, 24]. However, there is a dearth of knowledge surrounding the long-term effectiveness of such programmes, or adherence and effectiveness of the face-to-face counterparts [18, 19]. Hence, the main objective of the current study was to report on the long-term outcomes (24 and 48 weeks) of people with hip or knee OA participating in a digital self-management programme delivering first-line OA management, and statistically investigate the mean treatment effect of duration on pain and physical function, as well as differences in pain over time between hip and knee OA with or without additional covariates.*

***Item 3 page 4-5 excerpted text:** *Hence, the main objective of the current study was to report on the long-term outcomes (24 and 48 weeks) of people with hip or knee OA participating in a digital self-management programme delivering first-line OA management, and statistically investigate the mean treatment effect of duration on pain and physical function, as well as differences in pain over time between hip and knee OA with or without additional covariates.*

***Item 4:** *Please see the whole Methods section in the manuscript, page 5-8.*

***Item 5:** *Please see the whole Methods section in the manuscript, page 5-8.*

***Item 6a page 5-6, excerpted text:**

***Setting and participants***

*Participants joined the digital OA self-management and education programme (see Intervention below for details), through recommendation by their local orthopaedic surgeon or physiotherapist, and via online advertisements and campaigns placed on search engines and social networks. Included participants had a radiographic and or clinical diagnosis of hip or knee OA from a physical therapist or physician (about 95%). Individuals without a prior diagnosis had clinical OA confirmed by an orthopaedic surgeon or physiotherapist via telephone, or if deemed necessary were recommended to seek face-to-face care before inclusion in the programme. An e-questionnaire regarding the participants’ overall health as well as OA-related factors was completed at inclusion, together with a pain report (to be repeated weekly during the study) and a 30-second chair stand test (30CST) (to be repeated bi-weekly).*

*Data was extracted from the digital self-management programme registry on the 13th of March 2019. At that time point, the register contained data from 1709 Swedish participants that had reported one of their knees or hips as their most symptomatic joint (index joint), had been treated in the programme for at least three weeks with a minimum adherence of 70% and had registered ≥24 weeks before data extraction. Adherence was defined as the percentage of completed activities (exercises, text or video lessons on OA, and quizzes on lesson material) per the pre-defined period (the cut-off of 70% for the initial three weeks represents about 5 out of 7 days per week performing recommended activities), and mean adherence was defined as the group mean for the period of interest. Outcome analysis was made in two separate sub-samples; participants with a pain report from week 24 or adjacent week (+- 4 weeks) and week 48 or adjacent week (+- 6 weeks), respectively. Hence, if a pain report was missing at week 24 or 48, the report from the closest week available was used (if two pain reports were available at the two most adjacent weeks, e.g. week 25 and 23, mean pain for these two was used). Those included in the 48-week sub-sample were excluded from the 24-week sub-sample, to enable comparison.*

***Item 7 page 7-8, excerpted text:**

***Outcome measures***

*Joint pain was assessed at baseline, and weeks 12, 24 and 48 using the Numerical Rating Scale (NRS, discrete boxes 0-10) with the instruction [26]; Mark on this scale how much pain you had the last week in your hip/knee, followed by a 0-10 scale where 0 was defined as No pain and 10 was defined as Maximum pain. Minimal clinically important change (MCIC) of pain was defined as an improvement of 20% (slightly or moderately important improvement according to Tubach et al., 2012) [27]. As a measure of physical function, the 30CST from week 12, 24 and 48 was used [28], performed by the participant with the help of an instruction video with a coupled visual timer. The patient entered the performed number of repetitions after each test. Physical function data was handled similarly to the NRS, with week 24 ±4 adjacent weeks and week 48±6 adjacent weeks included for those with available NRS data. All outcomes were self-assessed, self-entered using the digital programme interface, and chosen based on the International Consortium for Health Outcomes Measurement Standard Set for Hip & Knee Osteoarthritis (ICHOM) [29].*

***Statistical analysis***

*Summary data are described by the mean value, standard deviation and number of observations or the number and percent of the categories of interest. Comparisons of baseline data between the 24-week sub-sample and excluded participants (with missing data due to ending treatment or not reporting pain) were performed using independent samples t-test and the Fisher’s Exact test (for dichotomous variables). The group-specific mean treatment effect of duration on pain and repetitions (30CST - physical function), as well as differences in pain over time between hip and knee OA with or without additional covariates, were estimated and tested using random slopes and intercepts models. Pain development over time was plotted for hip and knee OA, respectively.*

*To describe patients adhering for six months with contrasting pain severity at baseline, participants were divided into same-sized tertiles based on reported baseline pain, and mean pain per time point was calculated and plotted for each group (not performed for the 48-week sub-sample due to small numbers per group).*

*Significance level was set to p<0·05, and p-values and 95% confidence intervals were reported when applicable. Statistical calculations were performed in SPSS Version 25 (IBM Corporation, New York, USA) and Stata 15·1 (StataCorp LLC, Texas, USA).*

***Item 8 page 7, excerpted text:**

***Outcome measures***

*Joint pain was assessed at baseline, and weeks 12, 24 and 48 using the Numerical Rating Scale (NRS, discrete boxes 0-10) with the instruction [26]; Mark on this scale how much pain you had the last week in your hip/knee, followed by a 0-10 scale where 0 was defined as No pain and 10 was defined as Maximum pain. Minimal clinically important change (MCIC) of pain was defined as an improvement of 20% (slightly or moderately important improvement according to Tubach et al., 2012) [27]. As a measure of physical function, the 30CST from week 12, 24 and 48 was used [28], performed by the participant with the help of an instruction video with a coupled visual timer. The patient entered the performed number of repetitions after each test. Physical function data was handled similarly to the NRS, with week 24 ±4 adjacent weeks and week 48±6 adjacent weeks included for those with available NRS data. All outcomes were self-assessed, self-entered using the digital programme interface, and chosen based on the International Consortium for Health Outcomes Measurement Standard Set for Hip & Knee Osteoarthritis (ICHOM) [29].*

***Item 9 page 7-8, excerpted text:**

***Statistical analysis***

*Summary data are described by the mean value, standard deviation and number of observations or the number and percent of the categories of interest. Comparisons of baseline data between the 24-week sub-sample and excluded participants (with missing data due to ending treatment or not reporting pain) were performed using independent samples t-test and the Fisher’s Exact test (for dichotomous variables). The group-specific mean treatment effect of duration on pain and repetitions (30CST - physical function), as well as differences in pain over time between hip and knee OA with or without additional covariates, were estimated and tested using random slopes and intercepts models. Pain development over time was plotted for hip and knee OA, respectively.*

*To describe patients adhering for six months with contrasting pain severity at baseline, participants were divided into same-sized tertiles based on reported baseline pain, and mean pain per time point was calculated and plotted for each group (not performed for the 48-week sub-sample due to small numbers per group).*

*Significance level was set to p<0·05, and p-values and 95% confidence intervals were reported when applicable. Statistical calculations were performed in SPSS Version 25 (IBM Corporation, New York, USA) and Stata 15·1 (StataCorp LLC, Texas, USA).*

***Item 10 page 6, 8, excerpted text:** *Page 6:* *Data was extracted from the digital self-management programme registry on the 13th of March 2019. At that time point, the register contained data from 1709 Swedish participants that had reported one of their knees or hips as their most symptomatic joint (index joint), had been treated in the programme for at least three weeks with a minimum adherence of 70% and had registered ≥24 weeks before data extraction. Adherence was defined as the percentage of completed activities (exercises, text or video lessons on OA, and quizzes on lesson material) per the pre-defined period (the cut-off of 70% for the initial three weeks represents about 5 out of 7 days per week performing recommended activities), and mean adherence was defined as the group mean for the period of interest. Outcome analysis was made in two separate sub-samples; participants with a pain report from week 24 or adjacent week (+- 4 weeks) and week 48 or adjacent week (+- 6 weeks), respectively. Hence, if a pain report was missing at week 24 or 48, the report from the closest week available was used (if two pain reports were available at the two most adjacent weeks, e.g. week 25 and 23, mean pain for these two was used). Those included in the 48-week sub-sample were excluded from the 24-week sub-sample, to enable comparison.*

*Page 8: After identifying active users at week 24, a total of 920 individuals were found, whereof 290 individuals had not reported their pain into the register at or around week 24. Excluding those with 48-week data, 499 individuals reporting pain were included in the 24-week sub-sample. For the 48-week sub-sample, a total of 138 individuals with pain data at 48 weeks were included (Figure 1).*

***Item 11 page 7-8, excerpted text:**

***Statistical analysis***

*Summary data are described by the mean value, standard deviation and number of observations or the number and percent of the categories of interest. Comparisons of baseline data between the 24-week sub-sample and excluded participants (with missing data due to ending treatment or not reporting pain) were performed using independent samples t-test and the Fisher’s Exact test (for dichotomous variables). The group-specific mean treatment effect of duration on pain and repetitions (30CST - physical function), as well as differences in pain over time between hip and knee OA with or without additional covariates, were estimated and tested using random slopes and intercepts models. Pain development over time was plotted for hip and knee OA, respectively.*

*To describe patients adhering for six months with contrasting pain severity at baseline, participants were divided into same-sized tertiles based on reported baseline pain, and mean pain per time point was calculated and plotted for each group (not performed for the 48-week sub-sample due to small numbers per group).*

*Significance level was set to p<0·05, and p-values and 95% confidence intervals were reported when applicable. Statistical calculations were performed in SPSS Version 25 (IBM Corporation, New York, USA) and Stata 15·1 (StataCorp LLC, Texas, USA).*

***Item 12a-d page 7-8, excerpted text:**

***Statistical analysis***

*Summary data are described by the mean value, standard deviation and number of observations or the number and percent of the categories of interest. Comparisons of baseline data between the 24-week sub-sample and excluded participants (with missing data due to ending treatment or not reporting pain) were performed using independent samples t-test and the Fisher’s Exact test (for dichotomous variables). The group-specific mean treatment effect of duration on pain and repetitions (30CST - physical function), as well as differences in pain over time between hip and knee OA with or without additional covariates, were estimated and tested using random slopes and intercepts models. Pain development over time was plotted for hip and knee OA, respectively.*

*To describe patients adhering for six months with contrasting pain severity at baseline, participants were divided into same-sized tertiles based on reported baseline pain, and mean pain per time point was calculated and plotted for each group (not performed for the 48-week sub-sample due to small numbers per group).*

*Significance level was set to p<0·05, and p-values and 95% confidence intervals were reported when applicable. Statistical calculations were performed in SPSS Version 25 (IBM Corporation, New York, USA) and Stata 15·1 (StataCorp LLC, Texas, USA).*

***Item 13a-b page 8, excerpted text:** *After identifying active users at week 24, a total of 920 individuals were found, whereof 290 individuals had not reported their pain into the register at or around week 24. Excluding those with 48-week data, 499 individuals reporting pain were included in the 24-week sub-sample. For the 48-week sub-sample, a total of 138 individuals with pain data at 48 weeks were included (Figure 1).*

Please see Figure 1 in the manuscript.

***Item 15:** *Please see the whole Results section in the manuscript, page 8-12.*

***Item 16 page 11, excerpted text:**

***Treatment effect over time***

*The treatment effect was modelled in terms of monthly pain change and monthly change in repetitions as a function of treatment duration for both 24- and 48-week sub-samples using a mixed model with random slopes and intercepts. The models yielded marginally different estimates for pain with a decrease of -0·43 units (95% CI -0·51, -0·35) and -0·39 units (95% CI -0·43, -0·36) per month for the 24- and 48-week sub-sample, respectively. Similarly, physical function increased by 0·76 repetitions (95% CI 0·64, 0·89) and 0·72 repetitions (95% CI 0·65, 0·79) per month, for the 24- and 48-week sub-sample respectively. The group difference (comparing 24- and 48-week sub-samples) in pain change did not reach statistical significance.*

*BMI, index joint or gender did not importantly influence pain or physical function over time. Pain improvement was not affected by age, yet with increasing age, there was a statistically significant but not clinically relevant decrease in the improvement in physical function (-0·003 per year; 95% CI -0·005, -0·002; p<0·01). Mean and median values of pain and physical function, for each time point and per index joint and sub-sample, are reported in table 3.*

***Item 18 page 12, excerpted text:**

***Discussion***

*This is, to the best of our knowledge, the first study investigating the long-term benefits of patients adhering to a digital OA self-management programme. Results in two groups of 499 and 138 individuals being adherent to treatment for about 5 out of 7 days per week for 24 or 48 weeks showed a substantial reduction in the level of pain and an increase in physical performance per month of treatment. Based on available group level data, there were no signs of worsening of symptoms during participation. Around 70% of those undertaking the programme reached a clinically relevant pain reduction at both follow-ups, and could thus be characterized as responders to treatment, with no clear difference between people with knee or hip OA.*

***Item 19 page 14-15, excerpted text:**

*Some limitations of our study need to be addressed. The lack of a control group and randomization limit our ability to explain the mechanisms of the observed reduction in pain and increased physical function in these OA patients, or to claim a direct cause and effect relationship. However, previous systematic reviews and meta-analyses of controlled trials provide evidence of a cause and effect relationship between exercise and patient benefits [33, 34]. In the current study pain and physical function were both shown to improve over time. Taking this into consideration, and that patients of all pain levels at baseline improved, speaks against a major influence of the regression to the mean phenomenon related to the natural course of the disease explaining our results. Since results are based on a register of patients voluntarily choosing whether to report their outcomes and when to drop out of treatment, reflecting clinical reality, some data is missing. Although the results suggest that dropouts do not importantly differ from included participants in terms of descriptive factors at baseline, for future studies it would be of value to interview and follow those ending treatment, and their OA-related outcomes…..*

*…Finally, we could not control for other treatments undertaken by the participants during the study period, therefore use of concurrent non-pharmacological or pharmacological treatments may have influenced the results.*

***Item 20 page 15, excerpted text:**

*The challenges and barriers of delivering exercise and education based self-management programmes to the growing OA population are substantial. Considering the positive results showed in this and previous studies, digital interventions may represent a viable alternative for patients without access to or or not interested in participating in traditional face to face programmes. Digital interventions such as the present one may also complement traditional programmes to enhance long-term adherence to treatment.*

***Item 21 page 14-15, excerpted text:**

*Since results are based on a register of patients voluntarily choosing whether to report their outcomes and when to drop out of treatment, reflecting clinical reality, some data is missing. Although the results suggest that dropouts do not importantly differ from included participants in terms of descriptive factors at baseline, for future studies it would be of value to interview and follow those ending treatment, and their OA-related outcomes. The participant sample was drawn from a register and inclusion criteria comprised of an hip or knee OA diagnosis and having initiated treatment with a 70% adherence for the initial weeks, supporting generalisibility. Hence, the sample should reflect the population to a greater extent than randomized controlled trials that commonly have more stringent inclusion criteria.*

***Item 22 page 3, excerpted text:**

***Financial Disclosure Statement***

*Funding was received by LED from Vinnova - Sweden’s Innovation Agency (grant number: 2016-04187, www.vinnova.se) and Stiftelsen för Bistånd åt Rörelsehindrade i Skåne (grant number: 2019-01-20, www.stiftbistandskane.se) to the Department of Clinical Sciences Lund, Orthopaedics, Lund University, Sweden. In kind support (data gathering and extraction) was provided by Arthro Therapeutics Inc. The funders or supporter had no role in study design, data collection and analysis, decision to publish, or preparation of the manuscript.*
